# Independence of face identity and expression processing: exploring the role of motion

**DOI:** 10.3389/fpsyg.2015.00255

**Published:** 2015-03-13

**Authors:** Karen Lander, Natalie Butcher

**Affiliations:** ^1^School of Psychological Sciences, University of Manchester, Manchester, UK; ^2^School of Social Sciences, Business and Law, Teesside University, Middlesbrough, UK

**Keywords:** face identity, facial expression, independence, interdependence, motion

## Abstract

According to the classic Bruce and Young (1986) model of face recognition, identity and emotional expression information from the face are processed in parallel and independently. Since this functional model was published, a growing body of research has challenged this viewpoint and instead support an interdependence view. In addition, neural models of face processing emphasize differences in terms of the processing of changeable and invariant aspects of faces. This article provides a critical appraisal of this literature and discusses the role of motion in both expression and identity recognition and the intertwined nature of identity, expression and motion processing. We conclude by discussing recent advancements in this area and research questions that still need to be addressed.

## Introduction

A controversial issue in studies of face processing is whether facial identity and emotion are processed independently or interactively (see Posamentier and Abdi, 2003; Calder and Young, 2005). Early functional models of face recognition, like the Bruce and Young (1986) model, suggest that facial identity and emotional expression are processed in parallel and independently. However, there is evidence to support both the independence and interdependence of identity and expression processing.

## Independence between Identity and Expression Processing

Support for the independent parallel route viewpoint comes from different sources. Firstly, neuropsychological studies show double dissociations whereby some patients show impaired recognition of face identity (prosopagnosia) but not emotional expression, or vice versa (e.g., Kurucz and Feldmar, 1979; Bruyer et al., 1983; Tranel et al., 1988). Whilst these results are compelling, they may be biased by methodological difficulties (unusual methods of scoring, absence of control data; Calder and Young, 2005) or patients may adopt atypical strategies (see Adolphs et al., 2005).

Secondly, studies with non-impaired participants also provide some support for independence. For example, Young et al. (1986) found no difference in reaction times when making expression matching decisions to familiar and unfamiliar faces. Additionally Strauss and Moscovitch (1981) found that while face identity and expression perception both show Left Visual Field superiority, they could be differentiated in terms of overall processing time. Furthermore, Etcoff (1984) found evidence for independence using the Garner (1974) selective attention paradigm (but see later work outlined in Interdependence section).

Thirdly, studies using non-human primates have suggested that different cortical cell populations are sensitive to facial identity and facial expression (e.g., Perrett et al., 1984; Hasselmo et al., 1989; Hadj-Bouziane et al., 2008). This suggestion has also been supported in human studies using positron emission tomography (Sergent et al., 1994) and fMRI (Haxby et al., 2000; Winston et al., 2004). These findings are consistent (but not necessarily conclusive) with the idea of independent facial identification and expression processes.

## Interdependence between Identity and Expression Processing

Despite substantial evidence supporting the existence of dissociable systems, there are a growing number of studies suggesting that the processing of facial identity and emotional expression is interdependent (see Fitousi and Wenger, 2013 for review). In order to fully understand the dependence or independence of information processing during a given task, it is first important to know which information is required for that task. For example, to resolve the different tasks of identity and expression categorization (using the same stimulus), different face information is needed (e.g., Morrison and Schyns, 2001; Schyns et al., 2002). Before considering this issue, we outline more classic research on interdependence.

Schweinberger and Soukup (1998) suggested that an asymmetric relationship exists between identity processing and expression processing. Using the Garner (1974) selective attention paradigm, they found that the speed of identity classification judgments does not increase with irrelevant variations in expression, but the opposite is not the case (also see Schweinberger et al., 1999; Goshen-Gottstein and Ganel, 2000; Baudouin et al., 2002; Wang et al., 2013). In addition, previous work does not take into account the possibility that an interaction between dimensions could happen at the level of decision processes instead of perceptual representations. Multidimensional signal detection can be used to explore this issue (Fitousi and Wenger, 2013). Soto et al. (2015), using this technique found that the perception of emotional expressions was not affected by changes in identity, but the perception of identity was affected by changes in emotional expression. Thus, besides any decisional interactions arising from the data, emotional expression and identity were also perceptually interactive.

Interestingly, a “smiling” effect has been found whereby happy expressions impact identity judgements. Specifically, seeing smiling faces has been found to aid the recognition and/or encoding of identity (Kottoor, 1989) and the naming of famous faces (Gallegos and Tranel, 2005). Kaufmann and Schweinberger (2004) demonstrated that famous faces were recognized more quickly when displaying moderately positive expressions, relative to more intense happy or angry faces. Later work found reduced judgements of face familiarity for negative-expression faces, compared with neutral-expression or positive-expression faces (Lander and Metcalfe, 2007). These results support the notion of interdependence between expression and identity processing from faces.

More recent studies using an adaptation methodology further support this viewpoint. Results have shown that emotion aftereffects in individual expressions (when one of the target expressions matches the adapting face) are modulated by identity, with aftereffects in the same-identity condition larger than in the different-identity condition (Campbell and Burke, 2009; Vida and Mondloch, 2009). These results were taken as evidence for visual representations of expression faces that are both independent and dependent on identity (Fox and Barton, 2007; Ellamil et al., 2008; Pell and Richards, 2013).

Finally, computational work has also supported the possible overlap between representations of identity and expression (see Calder et al., 2001; Calder and Young, 2005) and imaging studies have found overlap in activation patterns during identity and facial expression recognition tasks (e.g., LaBar et al., 2003; Ganel et al., 2005). These converging results suggest an effect of facial expression on recognition, and disagree with the original Bruce and Young (1986) model, which proposes that changes in facial expressions should not influence identity recognition.

## Changeable and Invariant Aspects of Faces

Newer models of face perception refer to neural processing. Haxby et al. (2000) propose two functionally and neurologically distinct pathways to face analysis, the lateral pathway that preferentially responds to changeable aspects of faces (including expressions) and the ventral pathway that preferentially responds to invariant aspects of faces (identity). Visuo-perceptual representations of changeable facial aspects, including expressions, are thought to be mediated by the superior temporal sulcus, while visuo-perceptual representations of invariant characteristics of a face, like the recognition of identity, are coded by the lateral fusiform gyrus (Haxby et al., 2000). Here, as in the Bruce and Young (1986) functional account, independence is proposed between the processing of identity and expression, but a weaker anatomical (rather than functional) distinction is made between changeable (expression) and invariant (identity) aspects of face processing.

While it is clear that face expression processing can impact identity processing, almost all previous work has utilized static images as stimuli. Since faces are normally seen in motion, we argue that this approach is limiting. To demonstrate this issue, we first outline research looking at the impact of motion on face identity and expression processing, before assessing the intertwined nature of identity, expression and motion processing. Indeed, a familiar person’s characteristic facial expressions (for example, their wry smile) aids recognition of their identity, just as the unique structure of an individual’s face influences the way their emotions are expressed. Here, we note that facial expressions contain static and dynamic components. Similarly, when recognizing identity, a dynamic clip also contains static and dynamic components. Importantly, the dynamic component present in both expression and identity processing may be intrinsically linked and may involve the same information. We conclude by reviewing the questions that remain to be answered in this research area.

## Movement and the Recognition of Identity

Much previous research has assumed that only invariant aspects of the face provide identity relevant information. However, a substantial body of research has demonstrated that changeable aspects of a face also affect identity recognition. This effect is referred to as the “motion advantage” (e.g., Schiff et al., 1986; Knight and Johnston, 1997; Pike et al., 1997; Lander et al., 1999; O’Toole et al., 2002; Lander and Davies, 2007). A face can produce rigid or non-rigid motion. During rigid facial movements the face maintains its three-dimensional form, while the whole head changes its relative position and/or orientation. During non-rigid motion, individual parts of the face move in relation to one another, for example during speech/expressions. Both types of motion information are posited to be independent of identity processing in the Bruce and Young (1986) account, yet seeing a face move facilitates the encoding and recognition of facial identity (e.g., Hill and Johnston, 2001; Knappmeyer et al., 2003; Pilz et al., 2006). More specifically, non-rigid facial movement aids accurate and faster face matching (Thornton and Kourtzi, 2002); better learning of unfamiliar faces (Lander and Bruce, 2003; Butcher et al., 2011; rigid motion — Pike et al., 1997), and helps accurate identification of degraded familiar faces (Knight and Johnston, 1997; Lander et al., 2001).

Several theories have explained why movement facilitates identity recognition (O’Toole et al., 2002). Firstly, movement may allow people to build a better three-dimensional representation of the face and head via structure-from-motion processes (representation enhancement hypothesis); secondly, people may learn the characteristic motion patterns of the face and head of a person (supplemental information hypothesis); thirdly, the social cues carried in movement (emotional expressions, speech) may attract attention to the identity specific areas of the face, facilitating identity processing (social signals hypothesis).

Although findings of a movement advantage are robust, several studies have found that movement is primarily useful when static face recognition is impaired in some way (e.g., negation, Knight and Johnston, 1997; blurring, Lander et al., 2001). Interestingly, recent research has also demonstrated that developmental prosopagnosics are able to match, recognize and learn moving faces better than static ones (Steede et al., 2007; Longmore and Tree, 2013; Bennetts et al., 2015). Taken together, these findings suggest that changeable aspects of a face can constitute a useful supplementary cue for face recognition, particularly when recognition is impaired by degradation of stimuli or by perceiver impairment (also see Xiao et al., 2014).

## Movement and the Recognition of Expression

Similarly to identity research, past research on facial expression processing has utilized static facial images. However, expressions are changeable and dynamic in nature. Ordinarily people view dynamic facial expressions that make rapid changes over time, rather than static images of an expression “apex.” It is known that we are extremely sensitive to subtle dynamic cues (Edwards, 1998) and to changes of natural facial dynamics (Dobs et al., 2014). Furthermore, dynamic aspects (e.g., speed of onset/offset) of facial movement are useful when distinguishing genuine from posed expressions (Hess and Kleck, 1990) and often differences between expressions are reflected in their temporal dynamic properties (Ekman et al., 1985). Jack et al. (2014) propose that there are four basic emotional expressions, perceptually segmented across time. Furthermore, dynamic facial expressions are known to be recognized more accurately (Trautmann et al., 2009) and quickly (Recio et al., 2011, but see Fiorentini and Viviani, 2011) than static expressions (see Krumhuber et al., 2013 for review).

Further experimental evidence for the importance of dynamic information during expression recognition has been found in point-light experiments (Matsuzaki and Sato, 2008), experiments using subtle expressions (Ambadar et al., 2005) and those that impose time pressures (Zhongqing et al., 2014). Interestingly, Kamachi et al. (2001) found that the dynamic characteristics of the observed motion affected how well different morphed expressions could be recognized. Sadness was most accurately identified from slow sequences, with happiness and surprise, most accurately recognized from fast sequences. Angry expressions were best recognized from medium speed sequences and dynamic characteristics may be important in the “angry superiority effect” (Ceccarini and Caudek, 2013). Work by Pollick et al. (2003) found that changing the duration of an expression had an effect on ratings of emotional intensity, with a trend for expressions with shorter durations to have lower ratings of intensity (also see Bould and Morris, 2008). Finally, Gill et al. (2014) show that dynamic facial expressions override the social judgements made based on static face morphology.

In early work, Humphreys et al. (1993) report the case of a prosopagnosic patient who could make expression judgements from moving (but not static) displays, consistent with the idea of dissociable static and dynamic expression processing. Trautmann et al. (2009) used an fMRI methodology to examine the neural networks involved in the perception of static and dynamic facial expressions. Dynamic faces indicated enhanced emotion-specific brain activation patterns in the parahippocampal gyrus, including the amygdala, fusiform gyrus, superior temporal gyrus, inferior frontal gyrus, and occipital and orbitofrontal cortex. *Post hoc* ratings of the dynamic stimuli revealed a better recognisability in comparison to the static stimuli (but see Trautmann-Lengsfeld et al., 2013).

## Concluding Comments and Future Directions

Thus, the literature reviewed demonstrates that expression processing can impact face identification, and that movement more broadly influences both face identification and expression recognition. It seems plausible to suggest that this is because facial motion concurrently contains both identity-specific and expression information which, on an everyday basis, are processed simultaneously. Indeed, understanding the emotional facial expressions of others, and being able to identify those individuals are both important for daily social functioning. Typically a face moves in a complex manner, combining rigid rotational and non-rigid movements (O’Toole et al., 2002). However, in most studies investigating the role of motion in identity recognition, relatively unspecified speaking and expressive movements are utilized. Future research should systematically investigate the effect of different types of motion on both identity and expression recognition. In addition, it is difficult to separate out the impact of motion and expression, as it is possible that even seeing a static facial expression may activate the brain areas associated with producing that action ourselves. This notion is concordant with research that has found that the “classical” mirror neuron system (premotor and parietal areas), limbic regions, and the somatosensory system become spontaneously active during the monitoring of facial expressions and the production of similar facial expressions (van der Gaag et al., 2007). van der Gaag et al. (2007) used only moving stimuli, so it remains unclear whether similar mirror neuron activation is evident when the perceiver sees only the consequence of an expressive action (e.g., smiling action) in the form of a static expression (e.g., a smile). It is interesting to consider what additional questions remain in this rapidly progressing research area.

Firstly, given the importance of motion for the recognition of both identity and expressions, we need to determine whether neural models like Haxby et al. (2000) can account for the importance of motion when recognizing identity. This question is the focus of neuroimaging work that aims to determine the neural activities when processing moving and static faces (see Fox et al., 2009; Schultz and Pilz, 2009; Ichikawa et al., 2010; Pitcher et al., 2011a; Schultz et al., 2013). Indeed, recent research by Pitcher et al. (2014) suggests that the dynamic motor and static components of a face are processed via dissociable cortical pathways. Pitcher et al. (2014) revealed a double dissociation between the response to moving and static faces as thetaburst transcranial magnetic stimulation (TBS) delivered over the right occipital face area (OFA) reduced the response to static but not moving faces in the right posterior STS (rpSTS), while TBS delivered over the rpSTS itself reduced the response to dynamic but not static faces. Interestingly, they found that these dissociable pathways originate early in the visual cortex, not in the OFA, a finding that opposes prevailing models of face perception (Haxby et al., 2000; Calder and Young, 2005; Pitcher et al., 2011b), indicating that we may need to reconsider how faces are cortically represented.

A second issue concerns whether motion mediates the relationship between identity and expression processing. Stoesz and Jakobson (2013) used a speeded Garner paradigm task and found a difference between static and moving stimuli. There was no support for independence with static faces. However, when the faces were moving, participants’ identity and expression judgments were unaffected by modifications in the irrelevant dimension, supporting independence with moving faces. Moreover, using similar methods Rigby et al. (2013) found that dynamic facial information reduced the interference between upright facial identity and emotion processing. These findings indicate that static facial identity information and emotional information may interfere with one another. However, moving faces seem to promote the separation of facial identity and emotion expression processing. Future experimental work needs to investigate the role of motion in mediating the independence of identity and expression processing from faces, by specifically comparing independence using different methodologies with both static and moving stimuli.

A third issue links to the fact that in order to fully understand dependence or independence during a given task, it is first necessary to know which information is required for that task. It is known that different visual categorisation tasks (e.g., face identity, expression or gender) are sensitive to distinct visual characteristics of the same image (Schyns et al., 2002). For example, research suggests that a central band of spatial frequencies is particularly useful for identifying faces (e.g., Fiorentini et al., 1983; Parker and Costen, 1999). Specific methods (e.g., bubbles; Schyns et al., 2002) have been used to isolate information required for identity/expression recognition. Whilst some of the diagnostic cues required to identify an expression and a face may be distinct, others like facial motion may overlap. Similar methodologies should be adopted in the future to isolate what aspects of facial motion are diagnostic of face identity and expression.

A further issue concerns further distinctions that can be made regarding the type of motion shown by a face. Facial movements can involve expressions or not, and expressional movements may have a significant emotional content or have little affective content. In future work it may be possible to uncouple the impact of both expressional and non-expressional movement on the processing of facial identity. Furthermore, current findings may be modulated by other factors such as gender (see Herlitz and Lovén, 2013) or race (e.g., Hugenberg et al., 2007) and these should also be explored to gain a more representative understanding of the question (e.g., Henrich et al., 2010). For example, cultural specificities in static (Elfenbein and Ambady, 2003; Marsh et al., 2003) or dynamic facial expressions (Jack et al., 2012) may produce different patterns of information independence across cultures. Lastly, future neuroscience investigations are needed to probe the distinctive neural activities associated with moving face processing, focusing on expressional and non-expressional movements. These lines of enquiry will be important as they address how expression and identity processing are intertwined, and how motion mediates this relationship.

### Conflict of Interest Statement

The authors declare that the research was conducted in the absence of any commercial or financial relationships that could be construed as a potential conflict of interest.
